# Hospitalization costs of injury in elderly population in China: a quantile regression analysis

**DOI:** 10.1186/s12877-023-03729-0

**Published:** 2023-03-14

**Authors:** Wenjing Ou, Qin Zhang, Junlin He, Xinye Shao, Yang Yang, Xin Wang

**Affiliations:** 1 College of Health Management, China Medical University, Shenyang, 110122 Liaoning China; 2 Baotou Central Hospital, Baotou, 014040 Inner Mongolia China; 3 Shengjing Hospital of China Medical University, Shenyang, 110001 China; 4 Research Center for Health Development-Liaoning New Type Think Tank for University, China Medical University, Shenyang, 110122 Liaoning China

**Keywords:** Hospitalization costs, Injury, Elderly population, Quantile regression

## Abstract

**Background:**

Trauma in the elderly is gradually growing more prevalent as the aging population increases over time. The purpose of this study is to assess hospitalization costs of the elderly trauma population and analyze the association between those costs and the features of the elderly trauma population.

**Methods:**

In a retrospective analysis, data on trauma patients over 65 who were admitted to the hospital for the first time due to trauma between January 2017 and March 2022 was collected from a tertiary comprehensive hospital in Baotou. We calculated and analyzed the hospitalization cost components. According to various therapeutic approaches, trauma patients were divided into two subgroups: non-surgical patients (1320 cases) and surgical patients (387 cases). Quantile regression was used to evaluate the relationship between trauma patients and hospitalization costs.

**Results:**

This study comprised 1707 trauma patients in total. Mean total hospitalization costs per patient were ¥20,741. Patients with transportation accidents incurred the highest expenditures among those with external causes of trauma, with a mean hospitalization cost of ¥24,918, followed by patients with falls at ¥19,809 on average. Hospitalization costs were dominated by medicine costs (¥7,182 per capita). According to the quantile regression results, all trauma patients' hospitalization costs were considerably increased by length of stay, surgery, the injury severity score (16–24), multimorbidity, thorax injury, and blood transfusion. For non-surgical patients, length of stay, multimorbidity, and the injury severity score (16–24) were all substantially linked to higher hospitalization costs. For surgical patients, length of stay, injury severity score (16–24), and hip and thigh injuries were significantly associated with greater hospitalization costs.

**Conclusions:**

Using quantile regression to identify factors associated with hospitalization costs could be helpful for addressing the burden of injury in the elderly population. Policymakers may find these findings to be insightful in lowering hospitalization costs related to injury in the elderly population.

**Supplementary Information:**

The online version contains supplementary material available at 10.1186/s12877-023-03729-0.

## Background

Around 5.8 million people die from trauma each year, making it one of the major causes of death worldwide [1]. By 2030, there will be a rise in the number of injury-related fatalities worldwide, notably in low- and middle-income countries (LMICs) [2–4]. In 2019, China accounts for 18% of the world's population, with 164.5 million people aged 65 and older (65 +) and 26 million people aged 80 and older (80 +), and the proportion of people aged 65 and older is expected to be as high as 30% in 2050 [5, 6]. China has an aging population, and as it does, the strain on the country's current family and public healthcare systems will only become worse. Older folks' falls are a common and raising public health issue that can result in severe injuries (such as head injuries and hip fractures) [7]. It is estimated that approximately 30% of people aged 65 and over fall each year [8, 9] and an increasing number of elderly people suffer from hip fractures [10, 11]. By 2050, the total number of hip fractures in the elderly is expected to be 1.3 million in China [12]. Between 2015 and 2019, the older population is more likely to have a traffic injury, especially if they are over 45, and there is a rise in the mortality and morbidity linked to such injuries [13]. A study has demonstrated that multimorbidity is linked to increased health care utilization and out-of-pocket expenditure among the aged population over the age of 45 in China [14]. As a result, the above-described problems, which are primarily relevant to the elderly, will saddle China with a significant medical and budgetary burden in the future.

Previous studies on the cost of injury in the elderly population have focused more on falls [15–17], hip fractures [18–20] and head injuries [21, 22]. Traditional regression methods such as ordinary least squares (OLS), generalized linear models (GLM) and multiple linear regression have been mainly applied in previous cost studies [16, 17, 23], which are more concerned with overall mean effects, not robust to statistical outliers and lack flexibility in analyzing health care costs. Quantile regression (QR) models, which concentrate on the conditional quartiles of the cost distribution [24, 25], may be the best option for directing targeted cost-effective policies and early interventions, and can also give analysts the flexibility to examine trauma costs predictors corresponding to quantiles of interest.

In this study, we shed light on the hospitalization costs of the elderly trauma patients in China across the spectrum of injuries. Our aim is to assess the hospitalization costs for the trauma patients, non-surgical patients and surgical patients and to identify determinants of hospitalization costs through QR. In addition, understanding the magnitude of hospitalization costs for trauma patients aged ≥ 65 years is essential for providing evidence for cost-effective interventions and reference for health insurance policies.

## Methods

### Study population and data source

This is a retrospective study conducted in a tertiary comprehensive hospital in Baotou. Data is collected from the hospital information system (HIS) for each trauma patient episode from admission to hospital discharge. The following list of prerequisites applies:

Inclusion criteria:

(i) Patients who underwent their initial hospitalization for trauma between January 1, 2017 and March 31, 2022 were included.
(ii) Patients must have had a principal discharge diagnosis with the following International Classification of Diseases, 10th Revision (ICD-10), modification code: S00-T14 and an external cause of trauma with the code V01-Y98.
(iii) Patients are aged 65 or over.

Two trauma patients were excluded because the value of hospitalization cost was 0 and there were also incomplete information. The analysis comprised 1707 patients, who were divided into surgical (387 patients) and non-surgical (1320 patients) groups based on the type of treatments they received.

The data for patients’ medical records collected from HIS contained information on demographic characteristics, clinical data, and hospitalization costs.

### Study variables and outcome measures

#### Study variables

Based on research on the determinants impacting the cost of hospitalization for trauma patients, this study took into account a number of explanatory variables as follow:

1. Patients’ information comprising age, gender, and types of medical payments (basic medical insurance, entire self-pay and other);
2. Clinically relevant information, including length of stay (LOS), mode of admission, transferring to other departments, admission status, intensive care, admission to the intensive care unit (ICU), blood transfusion, rescue, usage of the ventilator, and therapeutic approaches;
3. Type of injury, cause of trauma, Injury Severity Score (ISS), Charlson Comorbidity Index (CCI), and multimorbidity are just a few of the parameters associated with injuries that can be found in the data. According to the site and kind of injury, trauma patients were divided into six categories: head injury, hip and thigh injury, abdominal, lower back, lumbar spine and pelvis injury, thorax injury, knee and lower leg injury, and others (see supplementary file 1). Trauma patients were divided into 5 categories according to the external cause of trauma, mostly including falls, transport accidents, exposure to inanimate mechanical forces, exposure to animate mechanical forces, and others (see supplementary file 2). The Abbreviated Injury Scale (AIS-90, update 2008) [26, 27] was used to generate the ISS, which was used to determine the total trauma severity. ISS scores were grouped into the following categories: Groups 1 (ISS < 16), 2 (16 ≤ ISS ≤ 24), and 3 (ISS ≥ 25). The severity of illnesses was evaluated using the Charlson comorbidity index (CCI) [28–30]. Multiple chronic noncommunicable diseases (NCDs) are referred to as multimorbidity [31, 32]. A total of 14 chronic diseases were included in the study and were used to calculate the number of NCDs for each patient. The chronic diseases included hypertension, diabetes, heart disease, stroke, chronic lung disease, asthma, cancer, osteoporosis, arthritis, liver disease, renal disease (excluding malignancy), digestive disorders, mental illness and memory related disorders.

#### Outcome variable

The total cost of hospitalization per admission served as the outcome variable. The sum of all subcategories of costs, such as diagnostic costs, treatment costs, surgical costs, and other costs-which are primarily broken down into the following groups: medicine costs, material costs, nursing costs, examination costs, general medical service costs, general treatment operation costs, surgical costs, and other costs-represents the total hospitalization costs. According to the consumer price index, all cost estimates were converted to 2017 RMB (¥) [33].

#### Statistical analyses

Categorical variables were presented as frequencies and their associated percentages.

Continuous variables were expressed as mean and standard deviation (SD). Two Independent sample t tests, the Wilcoxon rank-sum tests and the Chi-square tests were employed to test for differences in injury-related and hospitalization-related characteristics between non-surgical and surgical patients. The Wilcoxon rank sum test and the Kruskal–Wallis test were used to examine differences in mean costs across all patients for injury-related and hospitalization-related characteristics. According to the subgroup, we examined differences in mean costs in non-surgical patients and surgical patients, respectively. In this study, the ordinary least squares (OLS) and QR models were both applied. Multiple linear regression models were used to assess the overall effects of injury on hospitalization cost and quantile regression were used to estimate the effect of injury on hospitalization cost at the 10th, 25th, 50th, 75th and 90th percentiles. QR frequently employs median regression and estimates conditional quantile, while OLS approach focuses solely on conditional mean [34]. Therefore, QR is appropriate for assessing the consequences of cost-driving factors on the distribution of hospitalization expenditures as a whole. Outcome variable was log transformed before multivariable linear regression analysis and quantile regression.

We described the strength of the association between risk factors and hospitalization costs using coefficients and marginal effects with 95% CIs. The coefficients were determined using multiple linear regression and quantile regression after adjusting for all variables. Both surgical and non-surgical patients were included in our analysis of two subgroups. The coefficients at lower percentiles (10th and 25th percentiles) present the association between cost-driving factors and outcome on people whose hospitalization costs are low, while upper percentiles (75th and 90th percentiles) reflect the association on those whose hospitalization costs are higher.

Data analyses were employed using SAS version 9.4 (SAS Institute, Cary, NC). Stata12.0 was performed to generate charts. Statistical significance was based on two-sided tests and *P* values < 0.05 were considered statistically significant.

## Results

### Description of trauma patients and hospitalizations characteristics

The main characteristics of trauma patients and hospitalizations are shown in Table 1. A total of 1707 trauma patients were included in this study. The mean (SD) age of the trauma patients was 73.80 (6.92) years, with 53.54% of the male. The most common cause of trauma was falls (*n* = 901, 52.78%) and the most common type of injury was head injury (*n* = 657, 38.49%) followed by hip and thigh injuries (*n* = 303, 17.75%). The mean (SD) ISS was 10.46 (6.93), and 46.10% (*n* = 787) had two or more NCDs. The mean (SD) LOS was 15.49 (16.97) days. 387 patients (22.67%) underwent surgery. There were significant differences between surgical patients and non-surgical patients in terms of causes of trauma, types of injury and LOS.

**Table 1:** The main characteristics of trauma patients and hospitalizations

	**Total patients**	**Non-surgical patients**	**Surgical patients**	*P*
**Characteristics of hospitalization**				
**Total Hospitalizations, No. (%)**	1707	1320(77.33)	387(22.67)	
**Age, mean(SD), mo**	73.80(6.92)	73.98(6.97)	73.18(6.75)	0.1824
65–69	628(36.79)	469(35.53)	159(41.09)	
70–74	368(21.56)	282(21.36)	86(22.22)	
75–79	293(17.16)	238(18.03)	55(14.21)	
80–84	279(16.34)	217(16.44)	62(16.02)	
85–89	114(6.68)	95(7.2)	19(4.91)	
≥ 90	25(1.46)	19(1.44)	6(1.55)	
**Gender, No. (%)**				0.4712
Male	914(53.54)	713(54.02)	201(51.94)	
Female	793(46.46)	607(45.98)	186(48.06)	
**Medical Payment type, No. (%)**				0.0793
Basic Medical Insurance	861(50.44)	649(49.17)	212(54.78)	
Entire self-pay	489(28.65)	381(28.86)	108(27.91)	
Other	357(20.91)	290(21.97)	67(17.31)	
**Cause of injury, No. (%)**				< .0001
Falls (W00-W19)	901(52.78)	699(52.95)	202(52.2)	
Transport accidents (V01-V99)	528(30.93)	425(32.2)	103(26.61)	
Exposure to inanimate mechanical forces (W20-W49)	53(3.1)	22(1.67)	31(8.01)	
Exposure to animate mechanical forces (W50-W64)	31(1.82)	29(2.2)	2(0.52)	
Other	194(11.36)	145(10.98)	49(12.66)	
**Type of injury, No. (%)**				< .0001
Injuries to the head (S00-S09)	657(38.49)	577(43.71)	80(20.67)	
Injuries to the hip and thigh (S70-S79)	303(17.75)	199(15.08)	104(26.87)	
Injuries to the abdomen,lower back,lumbar spine and pelvis (S30-S39)	184(10.78)	154(11.67)	30(7.75)	
Injuries to the thorax (S20-S29)	179(10.49)	165(12.5)	14(3.62)	
Injuries to the knee and lower leg (S80-S89)	123(7.21)	68(5.15)	55(14.21)	
Other	261(15.29)	157(11.89)	104(26.87)	
**ISS, mean (SD)**	10.46(6.93)	10.37(6.74)	10.80(7.55)	0.1787
**ISS, No. (%)**				
< 16	1431(83.83)	1117(84.62)	314(81.14)	
16–24	228(13.36)	170(12.88)	58(14.99)	
≥ 25	48(2.81)	33(2.5)	15(3.88)	
**Multimorbidity**				0.3468
no NCDs	441(25.83)	338(25.61)	103(26.61)	
one NCDs	479(28.06)	374(28.33)	105(27.13)	
two NCDs	436(25.54)	347(26.29)	89(23)	
three or more NCDs	351(20.56)	261(19.77)	90(23.26)	
**CCI, mean (SD), mo**	3.83(1.62)	3.85(1.56)	3.76(1.82)	0.3288
≥ 3	1359(79.61)	1074(81.36)	285(73.64)	
< 3	348(20.39)	246(18.64)	102(26.36)	
**Characteristics of hospitalization**				
**LOS mean (SD), d**	15.49(16.97)	13.23(14.16)	23.20(22.59)	< .0001
≤ 7	618(36.2)	543(41.14)	75(19.38)	
8–14	451(26.42)	379(28.71)	72(18.60)	
15–28	422(24.72)	279(21.14)	143(36.95)	
≥ 29	216(12.65)	119(9.02)	97(25.06)	
**Mode of admission, No. (%)**				0.0248
Emergency admission	800(46.87)	638(48.33)	162(41.86)	
Outpatient admission	907(53.13)	682(51.67)	225(58.14)	
**Admission status, No. (%)**				0.2048
Critically ill status	45(2.64)	30(2.27)	15(3.88)	
Urgent status	204(11.95)	156(11.82)	48(12.40)	
General status	1458(85.41)	1134(85.91)	324(83.72)	
**Transferring to other departments**				< .0001
No	1491(87.35)	1253(94.92)	238(61.50)	
Yes	216(12.65)	67(5.08)	149(38.50)	
**Intensive care**				0.0057
No	1645(96.37)	1281(97.05)	364(94.06)	
Yes	62(3.63)	39(2.95)	23(5.94)	
**Admission to ICU**				< .0001
No	1580(92.56)	1292(97.88)	288(74.42)	
Yes	127(7.44)	28(2.12)	99(25.58)	
**Blood transfusion**				< .0001
No	1557(91.21)	1284(97.27)	273(70.54)	
Yes	150(8.79)	36(2.73)	114(29.46)	
**Rescue**				0.0298
No	1544(90.45)	1205(91.29)	339(87.60)	
Yes	163(9.55)	115(8.71)	48(12.40)	
**Usage of the ventilator**				< .0001
No	1552(90.92)	1270(96.21)	282(72.87)	
Yes	155(9.08)	50(3.79)	105(27.13)	
**In-hospital mortality**				0.4451
No	1643(96.25)	1268(96.06)	375(96.90)	
Yes	64(3.75)	52(3.94)	12(3.10)	

*SD* Standard Deviation, *ISS* Injury Severity Score, *NCDs* non-communicable diseases, *CCI* Charlson comorbidity index, *LOS* Length of stay, *ICU* Intensive care unit

*P*-value testing difference between non-surgical patients and surgical patients using Two Independent sample t tests, the Wilcoxon rank-sum tests and the Chi-square tests

### Summary of major cost factors by the type of treatments

**Fig. 1:**
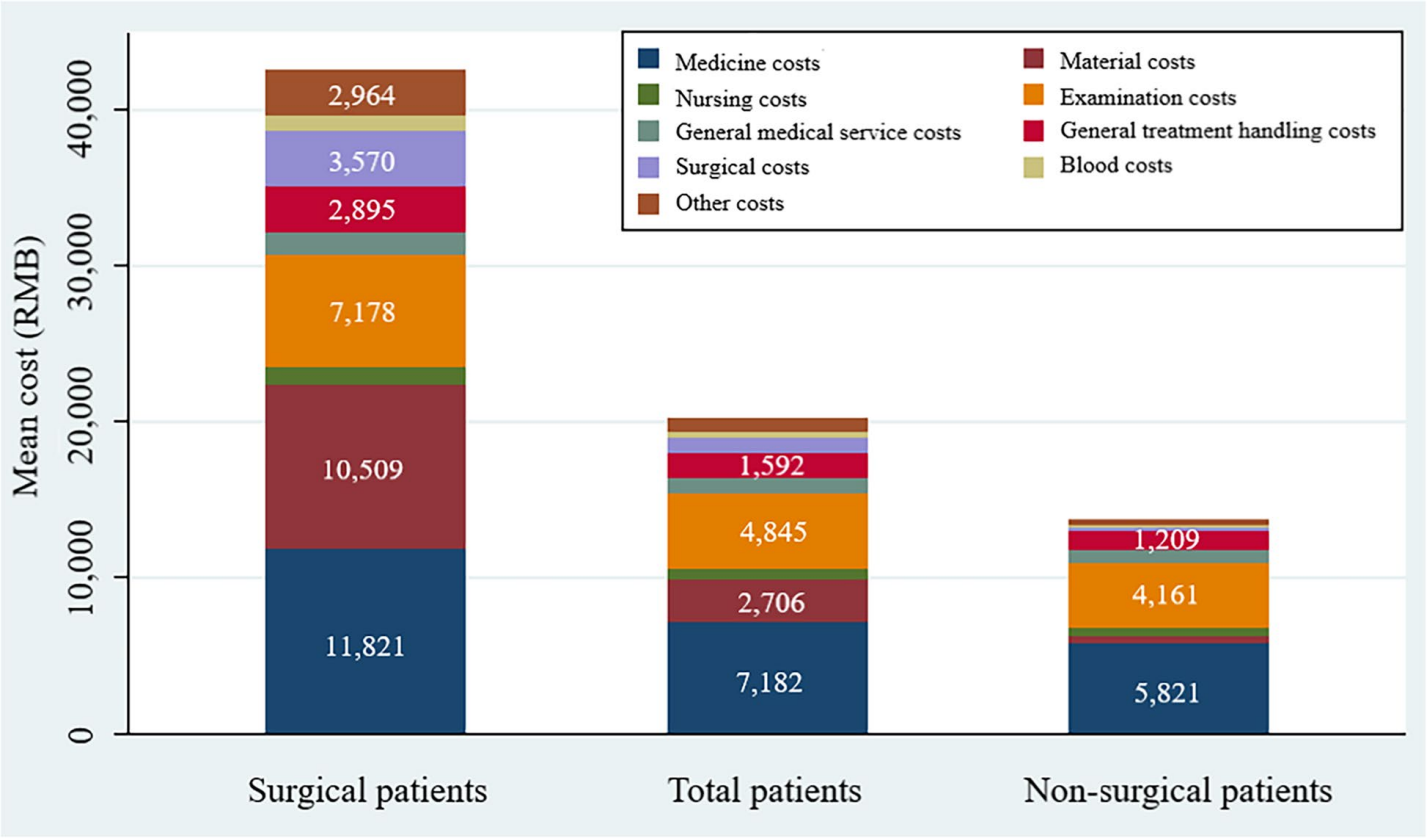
Mean hospitalization costs by the type of treatments. The horizontal coordinate indicates the classification of patients according to the type of treatment, and the vertical coordinate illustrates their average level according to the composition of hospitalization costs

Figure 1 shows Mean hospitalization costs by the type of treatments. Medicine cost was the main component of hospitalization cost. Patients undergoing surgery suffered the greatest average costs overall (medicine costs, material costs, nursing costs, examination costs, general medical service costs, general treatment handling costs, surgical costs, blood costs, and other costs). In addition to medicine costs, the highest cost category for surgical patients was material costs, and the highest cost category for non-surgical patients was examination costs. The cost of medicines as a percentage of overall hospitalization costs dropped from 41.93 percent to 26.34 percent between 2017 and 2022 (Z = -2.7649, *P* = 0.0057). The cost of the materials as a percentage of the total hospitalization costs received a boost from 11.01 percent to 18.87 percent (Z = 2.4348, *P* = 0.0149) (see additional file 3).

### Factors associated with hospitalization costs

Overall, mean hospitalization cost per patient was ¥20,741(SD ¥26,874) (Table 2). In terms of causes of trauma, the average hospitalization cost for traffic injury patients was ¥24,918 (SD ¥31,596), followed by falls (¥19,809) (SD ¥25,178)). Hip and thigh injuries had a mean hospitalization cost of ¥27,875 (SD ¥27,447). The average hospitalization cost increased with LOS from ¥6,415 for LOS ≤ 7 days to ¥62,407 for LOS ≥ 29 days. The mean hospitalization cost for surgical patients were higher than the average hospitalization cost for non-surgical patients (¥44,936 (SD¥36,471) VS ¥13,647 (SD ¥17,964)). For surgical patients, the average hospitalization costs for head injury, and hip and thigh injury were ¥59,751 (SD ¥53,613) and ¥56,839 (SD ¥20,518) respectively.

**Table 2:** Mean hospitalization Costs by trauma patients and hospitalizations in 2017 RMB (¥)

	**Total patients, Mean (SD)**	*P*	**Non-surgical patients, Mean (SD)**	*P*	**Surgical patients, Mean (SD)**	*P*
**Total**	20,741(26,874)		13,647(17,964)		44,936(36,471)	
**Age**		0.7397		0.8847		0.1507
65–69	21,088(28,622)		13,215(15,553)		36,289(27,789)	
70–74	19,480(23,207)		14,354(18,892)		35,108(26,838)	
75–79	21,452(27,970)		14,425(21,482)		51,860(32,335)	
80–84	20,112(22,882)		13,372(17,700)		43,706(23,438)	
85–89	18,787(29,242)		12,308(18,325)		51,181(47,803)	
≥ 90	20,346(22,095)		13,913(12,892)		40,717(33,009)	
**Gender**		0.0512		< .0001		0.2984
Male	21,913(28,189)		15,946(20,846)		43,080(38,772)	
Female	19,390(25,223)		10,947(13,353)		46,943(33,799)	
**Medical Payment type**		0.012		0.0006		0.0017
Basic Medical Insurance	18,825(23,332)		11,828(14,162)		40,243(31,488)	
Entire self-pay	22,719(27,313)		16,163(19,471)		45,848(36,916)	
Other	22,652(33,246)		14,411(22,546)		58,320(46,328)	
**Cause of injury**		< .0001		< .0001		< .0001
Falls (W00-W19)	19,809(25,178)		11,848(15,272)		47,354(32,335)	
Transport accidents (V01-V99)	24,918(31,596)		17,547(22,570)		55,331(43,358)	
Exposure to inanimate mechanical forces (W20-W49)	17,449(21,331)		12,946(18,481)		20,645(22,896)	
Exposure to animate mechanical forces (W50-W64)	8,509(18,560)		5,335(3,821)		54,530(73,468)	
Other	16,555(20,721)		12,656(14,047)		28,096(30,863)	
**Type of injury**		< .0001		0.0857		< .0001
Injuries to the head (S00-S09)	20,406(30,592)		14,951(20,659)		59,751(53,613)	
Injuries to the hip and thigh (S70-S79)	27,875(27,447)		12,738(16,097)		56,839(20,518)	
Injuries to the abdomen, lower back, lumbar spine and pelvis (S30-S39)	15,594(17,207)		12,407(12,418)		31,953(26,909)	
Injuries to the thorax (S20-S29)	14,213(17,919)		13,174(18,030)		26,453(10,987)	
Injuries to the knee and lower leg (S80-S89)	27,698(30,678)		15,935(21,271)		42,241(34,282)	
Other	18,128(22,255)		10,729(10,800)		29,297(29,396)	
**ISS**		< .0001		< .0001		< .0001
< 16	17,315(20,818)		11,439(12,932)		38,219(28,673)	
16–24	37,131(33,479)		24,014(29,676)		75,577(53,048)	
≥ 25	45,023(41,710)		34,994(40,088)		67,086(37,533)	
**Multimorbidity**		< .0001		< .0001		< .0001
no NCD	17,760(23,727)		11,253(13,635)		39,113(34,837)	
one NCD	16,549(20,623)		11,798(14,243)		33,471(29,274)	
two NCD	22,181(29,980)		14,180(18,966)		53,374(42,334)	
three or more NCD	28,418(31,900)		18,689(24,254)		56,633(34,652)	
**CCI**		0.5683		0.2777		0.5451
≥ 3	20,553(26,599)		13,904(18,789)		45,609(35,385)	
< 3	21,475(27,950)		12,525(13,776)		43,058(39,471)	
**LOS**		< .0001		< .0001		< .0001
≤ 7	6,415(6,754)		5,304(4,743)		14,457(11,871)	
8–14	13,925(12,281)		11,036(7,198)		29,133(20,025)	
15–28	27,677(19,973)		19,476(14,056)		43,678(20,148)	
≥ 29	62,407(44,463)		46,365(37,021)		82,089(45,084)	
**Mode of admission**		0.1526		0.0365		0.0517
Emergency admission	21,732(29,383)		14,728(20,863)		49,314(40,033)	
Outpatient admission	19,867(24,432)		12,636(14,690)		41,785(33,413)	
**Admission status**		< .0001		< .0001		< .0001
Critically ill status	56,439(60,273)		37,922(45,719)		93,473(69,883)	
Urgent status	29,114(35,802)		16,960(22,532)		68,614(42,170)	
General status	18,467(22,427)		12,549(15,311)		39,182(29,887)	
**Transferring to other departments**		< .0001		< .0001		< .0001
No	15,779(20,780)		12,552(16,141)		32,768(31,517)	
Yes	54,991(37,268)		34,128(32,533)		64,373(35,497)	
**Intensive care**		< .0001		< .0001		0.0098
No	19,901(26,385)		13,128(17,430)		43,736(36,742)	
Yes	43,030(30,206)		30,698(25,706)		63,940(25,723)	
**Admission to ICU**		< .0001		< .0001		< .0001
No	17,071(21,212)		12,673(14,644)		36,802(32,143)	
Yes	66,397(43,241)		58,607(58,052)		68,600(38,123)	
**Blood transfusion**		< .0001		< .0001		< .0001
No	16,763(21,544)		12,483(15,173)		36,893(32,805)	
Yes	62,031(39,244)		55,166(43,585)		64,199(37,717)	
**Rescue**		< .0001		< .0001		< .0001
No	18,616(22,354)		12,368(14,376)		40,822(30,187)	
Yes	40,872(48,942)		27,047(36,781)		73,993(58,196)	
**Usage of the ventilator**		< .0001		< .0001		< .0001
No	16,852(20,148)		12,413(13,794)		36,842(29,856)	
Yes	59,684(47,256)		45,003(52,123)		66,675(43,286)	
**In-hospital mortality**		0.0183		0.0197		0.0242
No	20,438(25,869)		13,414(17,154)		44,190(34,761)	
Yes	28,513(45,290)		19,340(31,654)		68,264(70,573)	

*SD* Standard Deviation, *ISS* Injury Severity Score, *NCDs* non-communicable diseases, *CCI* Charlson comorbidity index, *LOS L*ength of stay, *ICU* Intensive care unit

### Quantile regression

Table 3 describes the findings of quantile regression analysis of hospitalization costs for all trauma patients. Quantile regression analysis showed that LOS, ISS (16–24), having multimorbidity, thorax injuries, surgery and blood transfusions were significantly associated with higher hospitalization costs from the 10th percentile to the 90th percentile. The results suggest that the LOS is more obviously associated with greater hospitalization costs at the lower than upper quantiles, regardless of LOS at the 8–14, 15–28, and 29 levels. The effect of ISS (16–24) on the hospitalization costs distribution was greater at the lower than upper quantiles (coefficients 0.30, 95% CI 0.15–0.45 for 10th percentile; coefficients 0.18, 95% CI 0.06–0.29 for 90th percentile). Having multimorbidity and blood transfusion have a greater impact on hospitalization costs distribution at the lower than upper quantiles. The distribution of hospitalization costs at the higher quantiles was more impacted by surgery than at the lower quantiles. Thorax injuries exhibited a symmetrical trend in the distribution of hospitalization costs, and the effect values were constant at the 25th and 75th quartiles (coefficients 0.28 for 25th and 75th percentile).

**Table 3:** Multivariable quantile regression on hospitalization costs of the total trauma patients

	Quantile regression (Ln (Hospitalization costs))									
	10th percentile		25th percentile		50th percentile		75th percentile		90th percentile	
	coeff	95%CI	coeff	95%CI	coeff	95%CI	coeff	95%CI	coeff	95%CI
**Age (ref ≥ 90)**										
65–69	0.16	(-0.50–0.82)	0.11	(-0.20–0.42)	0.11	(-0.23–0.44)	-0.01	(-0.29–0.26)	-0.19	(-0.50–0.12)
70–74	0.34	(-0.30–0.98)	0.09	(-0.20–0.39)	0.05	(-0.28–0.38)	-0.02	(-0.29–0.25)	-0.17	(-0.48–0.14)
75–79	0.29	(-0.36–0.94)	0.09	(-0.20–0.39)	0.10	(-0.23–0.42)	0.05	(-0.24–0.33)	-0.11	(-0.41–0.20)
80–84	0.32	(-0.33–0.97)	0.1	(-0.18–0.39)	0.04	(-0.27–0.35)	-0.01	(-0.29–0.28)	-0.15	(-0.46–0.17)
85–89	0.25	(-0.43–0.93)	0.11	(-0.19–0.41)	0.06	(-0.30–0.42)	0.02	(-0.28–0.33)	-0.17	(-0.48–0.15)
**Gender: Male (ref = Female)**	0.11	(-0.01–0.22)	0.12**	(0.05–0.19)	0.11**	(0.04–0.18)	0.06*	(0.01–0.12)	0.10*	(0.02–0.18)
**Medical Payment type (ref = Other)**										
Basic Medical Insurance	0.14	(-0.02–0.30)	0.08	(-0.01–0.17)	0.03	(-0.08–0.13)	0.03	(-0.04–0.10)	0.05	(-0.06–0.16)
Entire self-pay	-0.13	(-0.29–0.04)	-0.11	(-0.22–0.00)	-0.13*	(-0.23–-0.03)	-0.02	(-0.11–0.08)	0.11	(-0.03–0.24)
**Cause of injury (ref = Other)**										
Falls (W00-W19)	0.05	(-0.12–0.22)	0.14*	(0.03–0.25)	0.12*	(0.00–0.23)	0.09	(-0.01–0.19)	0.13	(-0.02–0.28)
Transport accidents (V01-V99)	-0.04	(-0.22–0.14)	0.12*	(0.00–0.24)	0.07	(-0.05–0.18)	0.04	(-0.07–0.15)	0.08	(-0.07–0.23)
Exposure to inanimate mechanical forces (W20-W49)	-0.07	(-0.33–0.18)	-0.12	(-0.34–0.09)	-0.11	(-0.33–0.11)	-0.12	(-0.39–0.16)	-0.06	(-0.37–0.26)
Exposure to animate mechanical forces (W50-W64)	-0.28	(-0.71–0.15)	-0.35	(-0.76–0.06)	-0.1	(-0.37–0.17)	-0.31*	(-0.61–-0.00)	-0.33	(-0.95–0.28)
**Type of injury (ref = Other)**										
Injuries to the head (S00-S09)	0.04	(-0.13–0.22)	0.06	(-0.05–0.18)	-0.02	(-0.13–0.09)	-0.05	(-0.14–0.05)	0.02	(-0.12–0.16)
Injuries to the hip and thigh (S70-S79)	0.18	(-0.02–0.37)	0.19**	(0.06–0.33)	0.13*	(0.01–0.26)	0.14*	(0.03–0.25)	0.09	(-0.05–0.23)
Injuries to the abdomen, lower back, lumbar spine and pelvis (S30-S39)	0.18	(-0.06–0.43)	0.09	(-0.06–0.25)	0.03	(-0.14–0.20)	0.12	(-0.03–0.27)	0.16	(-0.02–0.33)
Injuries to the thorax (S20-S29)	0.21*	(0.01–0.42)	0.28***	(0.14–0.42)	0.25**	(0.09–0.41)	0.28***	(0.17–0.39)	0.21*	(0.04–0.39)
Injuries to the knee and lower leg (S80-S89)	0.21	(-0.03–0.45)	0.01	(-0.17–0.20)	0.07	(-0.07–0.21)	0.04	(-0.09–0.16)	-0.03	(-0.23–0.17)
**ISS (ref < 16)**										
16–24	0.30***	(0.15–0.45)	0.22**	(0.09–0.34)	0.32***	(0.21–0.43)	0.29***	(0.21–0.37)	0.18**	(0.06–0.29)
≥ 25	0.09	(-0.50–0.68)	0.1	(-0.22–0.41)	0.37***	(0.15–0.59)	0.41***	(0.18–0.64)	0.36*	(0.07–0.65)
**Multimorbidity (ref = no NCD)**										
one NCD	0.15*	(0.00–0.29)	0.13**	(0.03–0.22)	0.10*	(0.01–0.19)	0.08	(-0.01–0.16)	0.07	(-0.04–0.17)
two NCD	0.22**	(0.06–0.39)	0.26***	(0.15–0.36)	0.24***	(0.15–0.33)	0.16***	(0.07–0.24)	0.19**	(0.06–0.31)
three or more NCD	0.37***	(0.18–0.56)	0.40***	(0.28–0.51)	0.41***	(0.30–0.51)	0.33***	(0.23–0.43)	0.31***	(0.17–0.44)
**CCI ≥ 3 (ref < 3)**	-0.09	(-0.28–0.11)	0.07	(-0.21–0.07)	-0.06	(-0.17–0.04)	-0.06	(-0.17–0.06)	-0.06	(-0.21–0.10)
**LOS (ref ≤ 7)**										
8–14	0.92***	(0.78–1.06)	0.87***	(0.78–0.95)	0.74***	(0.66–0.83)	0.74***	(0.66–0.81)	0.64***	(0.53–0.74)
15–28	1.41***	(1.25–1.56)	1.38***	(1.27–1.48)	1.27***	(1.18–1.35)	1.23***	(1.14–1.31)	1.13***	(1.03–1.24)
≥ 29	1.92***	(1.70–2.15)	1.86***	(1.71–2.01)	1.87***	(1.72–2.02)	1.92***	(1.81–2.03)	1.84***	(1.69–2.00)
**Mode of admission: Emergency admission (ref = Outpatient admission)**	0.11*	(0.02–0.21)	0.06	(-0.01–0.13)	0.03	(-0.03–0.09)	-0.03	(-0.09–0.03)	-0.06	(-0.13–0.01)
**Admission status (ref = General status)**										
Critically ill status	-0.11	(-0.65–0.43)	-0.03	(-0.30–0.25)	-0.01	(-0.22–0.21)	-0.08	(-0.45–0.30)	0.22	(-0.23–0.67)
Urgent status	-0.01	(-0.17–0.15)	-0.01	(-0.11–0.09)	-0.09	(-0.20–0.02)	-0.04	(-0.13–0.05)	0.03	(-0.12–0.19)
**Transferring to other departments, Yes (ref = No)**	0.19	(-0.01–0.39)	0.21*	(0.05–0.38)	0.25***	(0.12–0.37)	0.15*	(0.02–0.27)	0.16*	(0.03–0.30)
**Intensive care, Yes (ref = No)**	0.70***	(0.47–0.93)	0.50***	(0.30–0.71)	0.47***	(0.33–0.61)	0.35***	(0.22–0.48)	0.24	(-0.07–0.54)
**Admission to ICU, Yes (ref = No)**	0.07	(-0.27–0.40)	0.05	(-0.16–0.25)	-0.08	(-0.27–0.11)	0.07	(-0.12–0.26)	0.08	(-0.20–0.36)
**Blood transfusion, Yes (ref = No)**	0.43**	(0.11–0.75)	0.26**	(0.07–0.45)	0.27***	(0.15–0.39)	0.15**	(0.04–0.25)	0.23**	(0.06–0.40)
**Rescue, Yes (ref = No)**	0.03	(-0.23–0.30)	0.08	(-0.10–0.26)	0.22*	(0.05–0.39)	0.30***	(0.17–0.43)	0.24**	(0.07–0.41)
**Usage of the ventilator, Yes (ref = No)**	0.29	(-0.02–0.59)	0.26*	(0.03–0.49)	0.26**	(0.08–0.44)	0.28**	(0.10–0.45)	0.30*	(0.05–0.56)
**In-hospital mortality, Yes (ref = No)**	0.04	(-0.50–0.58)	0.07	(-0.29–0.42)	0.14	(-0.14–0.42)	0.18	(-0.03–0.39)	0.15	(-0.10–0.39)
**Surgery, Yes (ref = No)**	0.47***	(0.30–0.65)	0.64***	(0.52–0.77)	0.59***	(0.49–0.69)	0.63***	(0.55–0.72)	0.66***	(0.56–0.77)

Coefficients estimated after adjusting for all variables in the table

*Ref* Reference, *Coeff* coefficient, *95%CI* 95%Confidence Interval, *ISS* Injury Severity Score, *NCDs* non-communicable diseases, *CCI* Charlson comorbidity index, *LOS* Length of stay, *ICU* Intensive care unit

^*^p-value < 0.05, **p-value < 0.01, ***p-value < 0.001

Table 4 illustrates the findings of quantile regression analysis of hospitalization costs for non-surgical patients. According to Quantile regression analysis, ISS (16–24), having multimorbidity, and LOS were linked to an increase in hospitalization costs across all the quartiles. The distribution of hospitalization costs at the lower quantiles is more affected by ISS (16–24), multimorbidity, and LOS than at the upper quantiles.

**Table 4:** Multivariable quantile regression on hospitalization costs of non-surgical patients

	Quantile regression (Ln (Hospitalization costs))									
	10th percentile		25th percentile		50th percentile		75th percentile		90th percentile	
	coeff	95%CI	coeff	95%CI	coeff	95%CI	coeff	95%CI	coeff	95%CI
**Age (ref ≥ 90)**										
65–69	0.25	(-0.77–1.26)	0.07	(-0.30–0.44)	-0.11	(-0.48–0.27)	-0.19	(-0.53–0.15)	-0.42*	(-0.74–-0.10)
70–74	0.34	(-0.65–1.33)	0.09	(-0.28–0.46)	-0.16	(-0.54–0.23)	-0.20	(-0.53–0.12)	-0.35*	(-0.67–-0.02)
75–79	0.34	(-0.65–1.34)	0.07	(-0.28–0.43)	-0.14	(-0.53–0.24)	-0.13	(-0.48–0.22)	-0.30	(-0.62–0.02)
80–84	0.31	(-0.69–1.31)	0.08	(-0.29–0.46)	-0.16	(-0.55–0.23)	-0.17	(-0.51–0.17)	-0.28	(-0.60–0.05)
85–89	0.25	(-0.81–1.31)	0.10	(-0.30–0.50)	-0.20	(-0.62–0.22)	-0.20	(-0.56–0.16)	-0.31	(-0.64–0.02)
**Gender: Male (ref = Female)**	0.08	(-0.04–0.20)	0.15***	(0.06–0.24)	0.17***	(0.10–0.24)	0.10**	(0.04–0.17)	0.14**	(0.05–0.22)
**Medical Payment type(ref = Other)**										
Basic Medical Insurance	0.29***	(0.12–0.46)	0.11	(-0.00–0.22)	0.06	(-0.05–0.16)	0.00	(-0.09–0.09)	0.06	(-0.05–0.16)
Entire self-pay	-0.08	(-0.26–0.10)	-0.12*	(-0.24–-0.00)	-0.14*	(-0.24–-0.03)	-0.08	(-0.19–0.03)	0.04	(-0.09–0.17)
**Cause of injury (ref = Other)**										
Falls (W00-W19)	-0.07	(-0.27–0.12)	0.10	(-0.03–0.23)	0.09	(-0.05–0.22)	0.05	(-0.07–0.17)	-0.04	(-0.18–0.10)
Transport accidents (V01-V99)	-0.01	(-0.22–0.19)	0.09	(-0.05–0.23)	0.03	(-0.10–0.17)	0.00	(-0.14–0.13)	0.04	(-0.12–0.20)
Exposure to inanimate mechanical forces (W20-W49)	-0.15	(-0.86–0.56)	-0.14	(-0.55–0.27)	-0.05	(-0.42–0.32)	0.05	(-0.07–0.17)	0.09	(-0.46–0.64)
Exposure to animate mechanical forces (W50-W64)	-0.41	(-0.90–0.08)	-0.3	(-0.74–0.14)	-0.12	(-0.40–0.17)	0.17	(-0.31–0.64)	-0.07	(-0.66–0.52)
**Type of injury (ref = Other)**										
Injuries to the head (S00-S09)	-0.08	(-0.28–0.12)	0.03	(-0.11–0.16)	-0.09	(-0.21–0.03)	-0.05	(-0.16–0.06)	0.05	(-0.09–0.18)
Injuries to the hip and thigh (S70-S79)	-0.07	(-0.33–0.19)	0.12	(-0.06–0.29)	0.04	(-0.12–0.19)	0.10	(-0.02–0.23)	0.14	(-0.02–0.29)
Injuries to the abdomen, lower back, lumbar spine and pelvis (S30-S39)	-0.01	(-0.24–0.23)	0.13	(-0.03–0.29)	-0.01	(-0.18–0.15)	0.11	(-0.05–0.27)	0.13	(-0.02–0.27)
Injuries to the thorax (S20-S29)	0.09	(-0.12–0.31)	0.22**	(0.06–0.39)	0.14	(-0.01–0.29)	0.23***	(0.11–0.36)	0.17*	(0.02–0.32)
Injuries to the knee and lower leg (S80-S89)	-0.01	(-0.36–0.35)	0.22	(-0.05–0.50)	0.03	(-0.13–0.20)	0.00	(-0.15–0.16)	-0.01	(-0.24–0.22)
**ISS (ref < 16)**										
16–24	0.30**	(0.09–0.50)	0.20**	(0.06–0.34)	0.29***	(0.17–0.41)	0.25***	(0.16–0.34)	0.15*	(0.03–0.26)
≥ 25	0.39	(-0.47–1.25)	0.23	(-0.13–0.60)	0.28	(-0.07–0.63)	0.41**	(0.13–0.69)	0.17	(-0.32–0.66)
**Multimorbidity (ref = no NCD)**										
one NCD	0.26**	(0.09–0.42)	0.20***	(0.10–0.30)	0.10*	(0.00–0.20)	0.11*	(0.02–0.20)	0.15*	(0.04–0.27)
two NCD	0.25**	(0.09–0.42)	0.27***	(0.15–0.39)	0.21***	(0.12–0.30)	0.16**	(0.06–0.26)	0.18**	(0.06–0.30)
three or more NCD	0.42***	(0.23–0.60)	0.45***	(0.30–0.60)	0.43***	(0.31–0.55)	0.38***	(0.27–0.50)	0.38***	(0.27–0.49)
**CCI ≥ 3 (ref < 3)**	-0.02	(-0.22–0.19)	-0.02	(-0.18–0.14)	-0.03	(-0.16–0.10)	-0.03	(-0.14–0.08)	-0.1	(-0.22–0.04)
**LOS (ref ≤ 7)**										
8–14	0.94***	(0.80–1.08)	0.87***	(0.77–0.96)	0.72***	(0.64–0.81)	0.72***	(0.64–0.79)	0.68***	(0.59–0.77)
15–28	1.43***	(1.28–1.59)	1.39***	(1.27–1.50)	1.28***	(1.20–1.37)	1.27***	(1.17–1.38)	1.29***	(1.14–1.44)
≥ 29	1.99***	(1.74–2.23)	1.95***	(1.78–2.12)	2.04***	(1.88–2.21)	1.95***	(1.81–2.09)	1.99***	(1.81–2.16)
**Mode of admission: Emergency admission (ref = Outpatient admission)**	0.09	(-0.02–0.21)	0.09*	(0.01–0.17)	0.07	(-0.00–0.14)	-0.02	(-0.08–0.05)	0.01	(-0.07–0.10)
**Admission status (ref = General status)**										
Critically ill status	-0.14	(-1.14–0.87)	-0.05	(-0.37–0.27)	-0.11	(-0.38–0.15)	-0.16	(-0.56–0.24)	0.10	(-0.39–0.60)
Urgent status	-0.05	(-0.25–0.15)	-0.03	(-0.16–0.09)	-0.07	(-0.18–0.04)	0.00	(-0.11–0.12)	0.05	(-0.10–0.20)
**Transferring to other departments, Yes (ref = No)**	0.12	(-0.15–0.39)	0.18	(-0.03–0.40)	0.18	(-0.01–0.36)	0.25**	(0.08–0.43)	0.21	(-0.02–0.45)
**Intensive care, Yes (ref = No)**	0.79***	(0.38–1.20)	0.68***	(0.48–0.89)	0.55***	(0.41–0.68)	0.38***	(0.23–0.53)	0.17	(-0.26–0.59)
**Admission to ICU, Yes (ref = No)**	0.32	(-0.37–1.01)	0.07	(-0.28–0.42)	0.08	(-0.28–0.43)	0.23	(-0.29–0.74)	0.56*	(0.01–1.12)
**Blood transfusion, Yes (ref = No)**	0.84***	(0.43–1.24)	0.66***	(0.47–0.85)	0.40***	(0.25–0.56)	0.34*	(0.04–0.64)	0.25	(-0.14–0.65)
**Rescue, Yes (ref = No)**	-0.01	(-0.34–0.33)	0.12	(-0.15–0.38)	0.22*	(0.00–0.44)	0.32***	(0.15–0.50)	0.27***	(0.13–0.40)
**Usage of the ventilator, Yes (ref = No)**	0.04	(-0.61–0.69)	0.54**	(0.14–0.94)	0.47**	(0.19–0.75)	0.41*	(0.08–0.75)	0.35	(-0.03–0.72)
**In-hospital mortality, Yes (ref = No)**	0.07	(-0.55–0.70)	-0.29	(-0.73–0.15)	-0.08	(-0.44–0.28)	0.03	(-0.27–0.33)	0.16	(-0.08–0.41)

Coefficients estimated after adjusting for all variables in the table

*Ref* Reference, *Coeff* coefficient, *95%CI* 95%Confidence Interval, *ISS* Injury Severity Score, *NCDs* non-communicable diseases, *CCI* Charlson comorbidity index, *LOS* Length of stay, *ICU* Intensive care unit

^*^p-value < 0.05, **p-value < 0.01, ***p-value < 0.001

The findings of the quantile regression analysis of hospitalization costs for surgical patients are presented in Table 5. According to quantile regression analysis, hospitalization costs were higher across all quantiles for ISS (16–24), LOS, and hip and thigh injuries. Hip and thigh injuries, LOS (15–28 and 29 levels), and ISS (16–24) all had a greater impact on the distribution of hospitalization costs at the lower quantiles than at the upper quantiles. Except for the 90th quantile, falls that were associated with higher hospitalization costs were significant in almost all quantiles. The effect of falls was more pronounced for the lower percentiles (coefficients 0.50, 95% CI 0.15–0.86 for the 10th percentile), compared with the upper percentiles (coefficients 0.23, 95% CI 0.00–0.46 for the 75th percentile).

**Table 5:** Multivariable quantile regression on hospitalization costs of surgical patients

	Quantile regression (Ln (Hospitalization costs))									
	10th percentile		25th percentile		50th percentile		75th percentile		90th percentile	
	coeff	95%CI	coeff	95%CI	coeff	95%CI	coeff	95%CI	coeff	95%CI
**Age (ref ≥ 90)**										
65–69	0.3	(-0.72–1.33)	0.09	(-0.53–0.72)	0.29	(-0.12–0.71)	0.19	(-0.32–0.70)	0.39	(-0.46–1.24)
70–74	0.33	(-0.75–1.40)	0.03	(-0.57–0.63)	0.13	(-0.27–0.53)	0.21	(-0.27–0.69)	0.30	(-0.54–1.14)
75–79	0.24	(-0.77–1.26)	0.06	(-0.54–0.66)	0.28	(-0.14–0.69)	0.24	(-0.25–0.74)	0.47	(-0.39–1.34)
80–84	0.04	(-0.96–1.03)	-0.01	(-0.59–0.57)	0.15	(-0.24–0.54)	0.11	(-0.42–0.63)	0.27	(-0.60–1.14)
85–89	0.5	(-0.78–1.78)	-0.03	(-0.77–0.71)	0.28	(-0.21–0.78)	0.26	(-0.24–0.77)	0.33	(-0.57–1.23)
**Gender: Male (ref = Female)**	-0.02	(-0.27–0.23)	-0.01	(-0.13–0.12)	-0.09	(-0.18–0.01)	-0.14*	(-0.26–-0.03)	-0.17*	(-0.31–-0.03)
**Medical Payment type (ref = Other)**										
Basic Medical Insurance	-0.27	(-0.61–0.07)	-0.05	(-0.26–0.16)	0	(-0.18–0.18)	-0.07	(-0.27–0.13)	-0.13	(-0.41–0.14)
Entire self-pay	-0.25	(-0.60–0.09)	-0.25*	(-0.48–-0.01)	0.09	(-0.13–0.31)	0.07	(-0.10–0.24)	-0.03	(-0.24–0.17)
**Cause of injury (ref = Other)**										
Falls (W00-W19)	0.50**	(0.15–0.86)	0.35**	(0.12–0.58)	0.27*	(0.05–0.48)	0.23*	(0.00–0.46)	0.10	(-0.13–0.33)
Transport accidents (V01-V99)	0.11	(-0.27–0.49)	0.20	(-0.09–0.48)	0.22	(-0.05–0.49)	0.13	(-0.11–0.37)	-0.08	(-0.40–0.24)
Exposure to inanimate mechanical forces (W20-W49)	0.43	(-0.13–0.99)	-0.07	(-0.41–0.27)	-0.01	(-0.29–0.28)	0.03	(-0.32–0.37)	-0.20	(-0.62–0.22)
Exposure to animate mechanical forces (W50-W64)	-0.16	(-5.10–4.77)	-0.79	(-3.52–1.93)	-0.59	(-3.13–1.95)	0.54	(-2.71–3.80)	-0.07	(-4.16–4.01)
**Type of injury (ref = Other)**										
Injuries to the head (S00-S09)	0.24	(-0.19–0.67)	0.23	(-0.03–0.49)	0.34**	(0.10–0.58)	0.22	(-0.02–0.46)	0.34*	(0.06–0.61)
Injuries to the hip and thigh (S70-S79)	0.81***	(0.37–1.25)	0.56***	(0.29–0.84)	0.42***	(0.22–0.63)	0.23*	(0.05–0.40)	0.23*	(0.03–0.42)
Injuries to the abdomen, lower back, lumbar spine and pelvis (S30-S39)	0.37	(-0.35–1.08)	0.11	(-0.26–0.47)	0.15	(-0.19–0.49)	0.20	(-0.16–0.57)	0.20	(-0.19–0.59)
Injuries to the thorax (S20-S29)	1.34*	(0.27–2.41)	0.85***	(0.41–1.29)	0.68***	(0.45–0.91)	0.31	(-0.19–0.81)	0.18	(-0.55–0.90)
Injuries to the knee and lower leg (S80-S89)	0.39	(-0.02–0.79)	0.08	(-0.20–0.35)	0.22	(-0.02–0.45)	0.16	(-0.05–0.37)	0.16	(-0.05–0.38)
**ISS (ref < 16)**										
16–24	0.76**	(0.29–1.23)	0.39**	(0.11–0.68)	0.33**	(0.10–0.57)	0.46***	(0.25–0.67)	0.35**	(0.11–0.58)
≥ 25	0.53	(-0.66–1.72)	0.48	(-0.15–1.11)	0.39*	(0.02–0.75)	0.48*	(0.10–0.85)	0.34	(-0.11–0.79)
**Multimorbidity (ref = no NCD)**										
one NCD	-0.31*	(-0.61–-0.01)	-0.14	(-0.36–0.07)	0.01	(-0.15–0.18)	-0.02	(-0.17–0.13)	0.05	(-0.13–0.24)
two NCD	0.02	(-0.37–0.41)	0.12	(-0.09–0.32)	0.16	(-0.01–0.32)	0.26**	(0.08–0.45)	0.26*	(0.01–0.51)
three or more NCD	0.15	(-0.17–0.48)	0.16	(-0.04–0.36)	0.20*	(0.04–0.35)	0.26**	(0.09–0.43)	0.26*	(0.03–0.49)
**CCI ≥ 3 (ref < 3)**	0.13	(-0.29–0.55)	0.00	(-0.25–0.25)	-0.03	(-0.21–0.16)	-0.13	(-0.31–0.06)	-0.12	(-0.35–0.12)
**LOS (ref ≤ 7)**										
8–14	0.65**	(0.25–1.05)	0.56***	(0.32–0.80)	0.76***	(0.54–0.97)	0.69***	(0.45–0.93)	0.58***	(0.29–0.87)
15–28	1.24***	(0.89–1.59)	1.08***	(0.87–1.29)	1.08***	(0.91–1.26)	0.96***	(0.74–1.18)	0.78***	(0.53–1.02)
≥ 29	1.51***	(0.99–2.03)	1.50***	(1.23–1.76)	1.53***	(1.29–1.76)	1.29***	(1.04–1.54)	1.23***	(0.89–1.56)
**Mode of admission: Emergency admission (ref = Outpatient admission)**	-0.01	(-0.20–0.19)	-0.03	(-0.16–0.10)	-0.06	(-0.16–0.04)	-0.05	(-0.16–0.07)	-0.14*	(-0.26–-0.01)
**Admission status (ref = Generalstatus)**										
Critically ill status	-0.08	(-1.12–0.97)	-0.07	(-0.69–0.54)	0.17	(-0.30–0.64)	0.42	(-0.16–0.99)	0.29	(-0.19–0.77)
Urgent status	0.18	(-0.16–0.51)	0.12	(-0.08–0.31)	-0.10	(-0.29–0.09)	-0.02	(-0.21–0.16)	0.07	(-0.24–0.39)
**Transferring to other departments, Yes (ref = No)**	-0.05	(-0.37–0.28)	0.16	(-0.03–0.35)	0.21*	(0.03–0.39)	0.24*	(0.05–0.44)	0.18	(-0.03–0.38)
**Intensive care, Yes (ref = No)**	0.30	(-0.15–0.75)	0.25	(-0.03–0.53)	0.11	(-0.11–0.32)	0.17	(-0.05–0.40)	-0.13	(-0.54–0.28)
**Admission to ICU, Yes (ref = No)**	0.45*	(0.02–0.88)	0.18	(-0.04–0.40)	0.15	(-0.03–0.33)	0.00	(-0.19–0.20)	0.10	(-0.11–0.31)
**Blood transfusion, Yes (ref = No)**	0.09	(-0.19–0.37)	0.13	(-0.04–0.31)	0.13*	(0.00–0.25)	0.08	(-0.06–0.22)	0.09	(-0.06–0.25)
**Rescue, Yes (ref = No)**	0.10	(-0.36–0.56)	0.12	(-0.12–0.36)	0.09	(-0.14–0.32)	0.06	(-0.22–0.33)	0.22	(-0.06–0.50)
**Usage of the ventilator, Yes (ref = No)**	-0.06	(-0.48–0.36)	-0.01	(-0.26–0.23)	0.06	(-0.11–0.21)	0.10	(-0.09–0.29)	0.02	(-0.20–0.23)
**In-hospital mortality, Yes (ref = No)**	0.13	(-1.39–1.64)	0.59	(-0.05–1.24)	0.37*	(0.04–0.70)	0.02	(-0.35–0.39)	-0.26	(-0.89–0.37)

Coefficients estimated after adjusting for all variables in the table

*Ref* Reference, *Coeff* coefficient, *95%CI* 95%Confidence Interval, *ISS* Injury Severity Score, *NCDs* non-communicable diseases, *CCI* Charlson comorbidity index, *LOS* Length of stay, *ICU* Intensive care unit

^*^p-value < 0.05, **p-value < 0.01, ***p-value < 0.001

### Multivariable linear regression

Table 6 shows the findings of multivariable regression analysis of hospitalization costs for total patients, non-surgical patients and surgical patients. Total patients' hospitalization costs were chiefly associated with LOS, ISS (16–24), having multimorbidity, thorax injuries, intensive care, emergency admission, blood transfusions and surgery. For non-surgical patients, male, having basic medical insurance, thorax injuries, ISS, having multimorbidity, LOS, transferring to other departments, intensive care, blood transfusions, rescue, and usage of the ventilator were linked to hospitalization costs. Hospitalization costs for surgical patients were associated with fall, head injuries, hip and thigh injuries, abdomen, lower back, lumbar spine and pelvis injuries, thorax injuries, ISS and LOS.

**Table 6:** Multivariable linear regression on hospitalization costs of total patients, non-surgical patients and surgical patients

	Multivariable linear regression (Ln (Hospitalization costs))					
	Total patients		Non-surgical patients		Surgical patients	
	coeff	95%CI	coeff	95%CI	coeff	95%CI
**Age (ref ≥ 90)**						
65–69	0.16	(-0.50–0.82)	-0.01	(-0.29–0.28)	0.46	(-0.02–0.94)
70–74	0.34	(-0.30–0.98)	0.01	(-0.27–0.29)	0.34	(-0.13–0.81)
75–79	0.29	(-0.36–0.94)	0.03	(-0.25–0.31)	0.45	(-0.03–0.92)
80–84	0.32	(-0.33–0.97)	0.00	(-0.28–0.28)	0.34	(-0.13–0.82)
85–89	0.25	(-0.43–0.93)	0.00	(-0.29–0.29)	0.37	(-0.15–0.90)
**Gender: Male (ref = Female)**	0.11	(-0.01–0.22)	0.13***	(0.06–0.20)	-0.12	(-0.24–0.00)
**Medical Payment type (ref = Other)**						
Basic Medical Insurance	0.14	(-0.02–0.30)	0.12*	(0.03–0.21)	-0.08	(-0.26–0.10)
Entire self-pay	-0.13	(-0.29–0.04)	-0.09	(-0.18–0.01)	-0.05	(-0.24–0.13)
**Cause of injury (ref = Other)**						
Falls (W00-W19)	0.05	(-0.12–0.22)	0.04	(-0.08–0.15)	0.29**	(0.10–0.47)
Transport accidents (V01-V99)	-0.04	(-0.22–0.14)	0.03	(-0.08–0.15)	0.16	(-0.06–0.37)
Exposure to inanimate mechanical forces (W20-W49)	-0.07	(-0.33–0.18)	-0.01	(-0.28–0.26)	-0.03	(-0.29–0.23)
Exposure to animate mechanical forces (W50-W64)	-0.28	(-0.71–0.15)	-0.22	(-0.46–0.02)	-0.17	(-0.99–0.65)
**Type of injury (ref = Other)**						
Injuries to the head (S00-S09)	0.04	(-0.13–0.22)	-0.02	(-0.13–0.10)	0.24*	(0.04–0.45)
Injuries to the hip and thigh (S70-S79)	0.18	(-0.02–0.37)	0.07	(-0.06–0.20)	0.44***	(0.24–0.64)
Injuries to the abdomen, lower back, lumbar spine and pelvis (S30-S39)	0.18	(-0.06–0.43)	0.05	(-0.09–0.18)	0.29*	(0.04–0.53)
Injuries to the thorax (S20-S29)	0.21*	(0.01–0.42)	0.18**	(0.05–0.32)	0.64***	(0.32–0.96)
Injuries to the knee and lower leg (S80-S89)	0.21	(-0.03–0.45)	0.03	(-0.14–0.19)	0.15	(-0.05–0.34)
**ISS (ref < 16)**						
16–24	0.30***	(0.15–0.45)	0.23***	(0.12–0.33)	0.36***	(0.15–0.57)
≥ 25	0.09	(-0.50–0.68)	0.23*	(0.02–0.45)	0.40*	(0.07–0.74)
**Multimorbidity (ref = no NCD)**						
one NCD	0.15*	(0.00–0.29)	0.15**	(0.06–0.25)	-0.11	(-0.27–0.06)
two NCD	0.22**	(0.06–0.39)	0.22***	(0.12–0.32)	0.14	(-0.05–0.32)
three or more NCD	0.37***	(0.18–0.56)	0.44***	(0.33–0.54)	0.13	(-0.07–0.32)
**CCI ≥ 3 (ref < 3)**	-0.09	(-0.28–0.11)	0.02	(-0.09–0.14)	0.03	(-0.17–0.23)
**LOS (ref ≤ 7)**						
8–14	0.92***	(0.78–1.06)	0.82***	(0.74–0.91)	0.65***	(0.46–0.84)
15–28	1.41***	(1.25–1.56)	1.37***	(1.28–1.46)	1.07***	(0.88–1.25)
≥ 29	1.92***	(1.70–2.15)	2.03***	(1.90–2.16)	1.56***	(1.34–1.77)
**Mode of admission: Emergency admission (ref = Outpatient admission)**	0.11*	(0.02–0.21)	0.03	(-0.04–0.10)	-0.04	(-0.16–0.08)
**Admission status (ref = General status)**						
Critically ill status	-0.11	(-0.65–0.43)	-0.07	(-0.30–0.15)	0.16	(-0.16–0.48)
Urgent status	-0.01	(-0.17–0.15)	-0.03	(-0.13–0.07)	0.01	(-0.18–0.20)
**Transferring to other departments, Yes (ref = No)**	0.19	(-0.01–0.39)	0.22**	(0.06–0.38)	0.14	(-0.03–0.31)
**Intensive care, Yes (ref = No)**	0.70***	(0.47–0.93)	0.52***	(0.33–0.72)	0.17	(-0.10–0.45)
**Admission to ICU, Yes (ref = No)**	0.07	(-0.27–0.40)	0.17	(-0.12–0.46)	0.20	(-0.02–0.42)
**Blood transfusion, Yes (ref = No)**	0.43**	(0.11–0.75)	0.49***	(0.27–0.72)	0.15	(-0.01–0.30)
**Rescue, Yes (ref = No)**	0.03	(-0.23–0.30)	0.19*	(0.03–0.35)	0.09	(-0.13–0.32)
**Usage of the ventilator, Yes (ref = No)**	0.29	(-0.02–0.59)	0.46***	(0.20–0.72)	0.03	(-0.18–0.24)
**In-hospital mortality, Yes (ref = No)**	0.04	(-0.50–0.58)	-0.11	(-0.33–0.11)	0.23	(-0.14–0.61)
**Surgery, Yes (ref = No)**	0.47***	(0.30–0.65)	-	-	-	-

Coefficients estimated after adjusting for all variables in the table

*Ref* Reference, *Coeff* coefficient, *95%CI* 95%Confidence Interval, *ISS* Injury Severity Score, *NCDs* non-communicable diseases, *CCI* Charlson comorbidity index, *LOS* Length of stay, *ICU* Intensive care unit

^*^p-value < 0.05, **p-value < 0.01, ***p-value < 0.001

## Discussion

Trauma has steadily developed into a global health issue in the elderly population as a result of population aging, with consequences such as disability and death. Our study uses QR to clarify the diverse impacts of influencing factors on hospitalization costs and primarily describes the features of the elderly trauma group over 65 years old. According to the results of our quantile regression analysis, trauma patients have greater hospitalization costs when they have LOS, surgery, ISS (16–24), multimorbidity, thorax injury, and blood transfusion. More importantly, our results suggest that the impacts of LOS, surgery, ISS (16–24), multimorbidity, thorax injury and blood transfusion on hospitalization costs were not constant across all the quantiles. Costlier hospitalizations for non-surgical patients are related to LOS, ISS (16–24), and multimorbidity. In-hospital expenses were particularly high in patients with significant injury and multimorbidity, according to a similar research conducted in the Netherlands [23]. Actually, it is not surprising that LOS and surgery were associated with higher hospitalization costs. The length of stay (LOS) is an indicator of quality of health care [35], and a rise in LOS indicates an increase in the consumption of medical resources. The expense of hospitalization could be decreased by lowering the LOS. On November 19, 2021, the National Healthcare Security Administration released the notice of the three-year action plan for the reform of the DRGs (Diagnosis Related Groups) and DIP (Diagnosis-Intervention Packet) payment modes [36]. The country-wide rollout of DRG and DIP is intended to optimize health resources, rationally regulate hospital costs, scale back on excessive LOS, and bolster the efficiency of the health insurance fund. In China, the LOS of C-DRGs (the Chinese diagnosis-related group) patients with hip fracture have been on the decline, according to a recent study on Sanming City [37]. Future research will continue to focus on the impact of DRGs/DIP on LOS without compromising quality of care, as shorter LOS may lead to inadequate treatment, more complications, or more readmissions.

Hospitalization costs are impacted differently by various therapeutic interventions. The average cost of hospitalization was ¥20,741 ($3,072) per patient. In contrast to the average hospitalization cost of €5430 ($6,134) in a Netherlands study [23], the average hospitalization cost for non-surgical patients was ¥13,647 ($2,021). In terms of surgical patients, average hospitalization costs for surgical patients are greater than for non-surgical patients (¥44,936 ($6,655) VS ¥13,647 ($2,021)). The quantile regression results illustrate significant associations of LOS, ISS (16–24), and hip and thigh injuries with higher hospitalization costs. And these effect values differed significantly across all quantiles. The average hospitalization cost for patients who underwent surgery for hip and thigh injuries was ¥56,839 ($8,418). Palais et al. showed that the mean hospitalization costs of patients who received hip replacement was $22,076 in 2018 [38]. In our study, the average hospitalization cost for all trauma patients with hip and thigh injuries was ¥27,875 ($4,129). According to a study conducted in Ireland in 2022, the mean hospitalization cost of patients with hip fractures was approximately €11,700 [19]. The cost of treating hip injuries differs across nations due to treatment practices, health insurance systems, hospital protocols and socio-demographic factors, however it is obvious that hip fractures incur significant medical expenses. The number of elderly people undergoing complex surgeries will rise as the population ages and medical technology continues to progress, and the entire healthcare system will face challenges in caring for these patients who may have high needs and high costs [39].

On the other hand, in contrast to ordinary least square regression, our study also discovered and confirmed that quantile regression, because of its capacity to evaluate each factor's impact at any point of the hospitalization costs distribution, can provide more detailed and comprehensive information on potential correlation when exploring the factors of hospitalization costs. Other factors like usage of the ventilator, rescue, transferring to other departments, intensive care, admission to ICU, and ISS(≥ 25) were associated with increased hospitalization costs in a specific or extreme (low or high) quantile. In addition to LOS, surgery, ISS (16–24), having multimorbidity, having a thorax injury, and receiving blood transfusions, other factors also indirectly reflect the severity of the patient's disease and the patient's critical status. This suggests that when applying DRGs and DIPs, it is more worthwhile for policy makers and researchers to consider not only the disease itself and the treatment of the disease, but also factors and specific information that indirectly affect hospitalization costs, such as the severity of injury in the elderly trauma population. Therefore, in order to more effectively lower hospitalization costs, the DRG/DIP payment standard may be dynamically adjusted as needed at any moment.

At the clinical level, reference to factors affecting hospitalization costs provides a theoretical basis for rational cost control. At the medical management level, consideration of hospitalization costs and influencing factors of the elderly trauma population provides some reference for relevant medical policy proposals.

In our study, we discovered that falls were the most common cause of trauma, and the average hospitalization cost for falls was ¥19,809 ($2,934), which was less expensive than the average hospitalization cost of $29, 562 for patients with nonfatal falls in the United States [40] and the range of $5,654 to $42,840 per fall-related hospitalization in the study by Heinrich et al. [41]. Age-related brain shrinkage affects the parts of older people's brains connected to physical activity [42, 43], and age-related losses in active muscle strength and cognitive function raise the risk of falls and fractures in older people. Additionally, traffic accidents are another major external cause of trauma. The majority of the elderly population in our study was more vulnerable to traffic accidents while walking or bicycling because they may have reduced peripheral vision, reaction activity, balance, and coordination [44, 45] and are more likely to have head and thorax injuries. Exercise instruction and routine screening for pertinent risk factors in the elderly population should be enhanced from a prevention aspect, and when needed, personalized fall prevention programs should be implemented [46]. Age-related pedestrian safety awareness should be improved by public health initiatives and safety education on the use of bicycle is necessary.

Our findings indicated that the major contributor to hospitalization costs was the cost of the medicine. In recent years, our country has implemented regulations to limit the medicine costs. As a result of the measures, the percentage of medicine costs has dropped from 41.93 percent to 26.34 percent, according to the findings of our study. Therefore, it is essential to further strengthen the supervision of rational medicine and standardize medication. We found a slight increase in the supplies cost. In order to cut medical costs and financial burdens for patients with fractures or trauma in the future, China is also actively developing and implementing centralized bulk procurement of high-value medical consumables.

To our knowledge, this is the first study in China to use quantile regression to examine the impacts of trauma on hospitalization costs in elderly population. This study also had several limitations. First of all, because the study group was restricted to a single hospital, it is impossible to extrapolate the findings to other areas due to potential bias caused by disparities in injury patterns and severity. More extensive studies are needed in the future. Secondly, we examined only hospitalization costs because hospitalization costs account for a significant portion of total injury costs. However, we study patients who are hospitalized for the first time for an injury, and some patients may be transferred to other hospitals without having their medical costs fully determined. This may underestimate the true total cost of the injury. Future studies should concentrate more on post-hospital health care costs and indirect costs (e.g., transportation, lost productivity) in order to fully assess the financial burden on the elderly trauma population. Thirdly, we only studied injuries based on anatomical sites, but the study of poisoning, and certain other consequences of external causes cannot be ignored. In the future studies, we further discuss their different categories of trauma and different causes of trauma separately. Finally, future studies could further expand the sample size to explore the financial burden of hospitalization costs for the elderly trauma population under different economic conditions.

## Conclusions

This study can effectively identify and measure the association between injuries and hospitalization costs at different quartiles in the elderly population by employing QR. Hospitalization costs in elderly patients with injuries were mainly associated with LOS, surgery, ISS (16–24), multimorbidity, thorax injury, and blood transfusions. LOS, ISS (16–24) and multimorbidity were positively associated with hospitalization costs for elderly non-surgical patients. Hospitalization costs for geriatric surgical patients were chiefly associated with LOS, ISS (16–24) and hip and thigh injuries. These findings provide support for scientific investigation of the factors influencing hospitalization costs and inform policy makers regarding the reduction of injury-related hospitalization costs in the elderly population.

## Supplementary Information

**Additional file 1. **Description of detailed composition of injury types.

**Additional file 2. **Description of detailed composition of causes of trauma.

**Additional file 3. **Distribution of hospitalization costs and their changing trends during 6 years.

**Additional file 4: Table S1.** Multivariable quantile regression on hospitalization costs of the total trauma patients: Adjusting for the time factor.

**Additional file 5: Table S2.** Multivariable quantile regression on hospitalization costs of non-surgical patients: Adjusting for the time factor.

**Additional file 6: Table S3.** Multivariable quantile regression on hospitalization costs of surgical patients: Adjusting for the time factor.

## Data Availability

The datasets generated and/or analysed during the current study are not publicly available due to ethical approval restrictions involving the inclusion of potentially identifying or sensitive patient data and anonymity but are available from the corresponding author on reasonable request.

## Abbreviations

LMICs: Low- and middle-income countries
OLS: Ordinary least squares
GLM: Generalized linear model
QR: Quantile regression
HIS: Hospital information system
ICD-10: International Classification of Diseases, 10th Revision
LOS: Length of Stay
ICU: Intensive Care Unit
ISS: Injury Severity Score
CCI: Charlson Comorbidity Index
AIS: Abbreviated Injury Scale
NCDs: Noncommunicable diseases
SD: Standard deviation
95% CI: 95% Confidence Interval
DRGs: Diagnosis Related Groups
DIP: Diagnosis-Intervention Packet
C-DRG: Chinese diagnosis-related group
