# Supplementary material for: Hospitalization costs of injury in elderly population in China: a quantile regression analysis
Source: BMC Geriatr. 2023 Mar 14;23:143. doi: 10.1186/s12877-023-03729-0 (PMC10013238; doi:10.1186/s12877-023-03729-0)
Supplement: Supplementary file 1 — Additional file 1. Description of detailed composition of injury types. [file 12877_2023_3729_MOESM1_ESM.pdf]

Additional file1. Description of detailed composition of injury types

Type of injury	ICD-10	Number of cases
Injuries to the head	S00-S09	657
Injuries to the hip and thigh	S70-S79	303
Injuries to the abdomen, lower back, lumbar spine and pelvis	S30-S39	184
Injuries to the thorax	S20-S29	179
Injuries to the knee and lower leg	S80-S89	123
Injuries to the shoulder and upper arm	S40-S49	75
Injuries to the elbow and forearm	S50-S59	46
Injuries to the ankle and foot	S90-S99	36
Injuries to the wrist and hand	S60-S69	35
Injuries to the neck	S10-S19	30
Injuries to unspecified parts of trunk, limb or body region	T08-T14	20
Injuries involving multiple body regions	T00-T07	19
Total		1707
