# Supplementary material for: Hospitalization costs of injury in elderly population in China: a quantile regression analysis
Source: BMC Geriatr. 2023 Mar 14;23:143. doi: 10.1186/s12877-023-03729-0 (PMC10013238; doi:10.1186/s12877-023-03729-0)
Supplement: Supplementary file 2 — Additional file 2. Description of detailed composition of causes of trauma. [file 12877_2023_3729_MOESM2_ESM.pdf]

Additional file 2. Description of detailed composition of causes of trauma

**Cause of injury**	**ICD-10**	**Number of cases**
Falls	W00-W19	901
Transport accidents	V01-V99	528
Exposure to inanimate mechanical forces	W20-W49	53
Exposure to animate mechanical forces	W50-W64	31
Other accidental threats to breathing	W75-W84	2
Exposure to forces of nature	X30-X39	1
Accidental poisoning by and exposure to noxious substances	X40-X49	1
Overexertion, travel and privation	X50-X57	5
Accidental exposure to other and unspecified factors	X58-X59	107
Intentional self-harm	X60-X84	7
Assault	X85-Y09	16
Event of undetermined intent	Y10-Y34	45
Sequelae of external causes of morbidity and mortality	Y85-Y89	1
Supplementary factors related to causes of morbidity and mortality classified elsewhere	Y90-Y98	7
**Falls**	W00-W19	901
Fall on same level from slipping,tripping and stumbling	W01	274
Other fall on same level due to collision with, or pushing by, another person	W03	2
Fall involving bed	W06	15
Fall involving chair	W07	8
Fall involving other furniture	W08	3
Fall on and from stairs and steps	W10	7
Fall on and from ladder	W11	4
Fall from, out of or through building or structure	W13	10

Additional file 2. Description of detailed composition of causes of trauma (*Continued*)

**Cause of injury**	**ICD-10**	**Number of cases**
Other fall from one level to another	W17	15
Other fall on same level	W18	31
Unspecified fall	W19	528
**Transport accidents**	V01-V99	528
Pedestrian injured in transport accident	V01-V09	184
Pedal cyclist injured in transport accident	V10-V19	80
Motorcycle rider injured in transport accident	V20-V29	18
Occupant of three-wheeled motor vehicle injured in transport accident	V30-V39	13
Car occupant injured in transport accident	V40-V49	4
Bus occupant injured in transport accident	V70-V79	4
Other land transport accidents	V80-V89	196
Other and unspecified transport accidents	V98-V99	29
