# Supplementary material for: Hospitalization costs of injury in elderly population in China: a quantile regression analysis
Source: BMC Geriatr. 2023 Mar 14;23:143. doi: 10.1186/s12877-023-03729-0 (PMC10013238; doi:10.1186/s12877-023-03729-0)
Supplement: Supplementary file 3 — Additional file 3. Distribution of hospitalization costs and their changing trends during 6 years. [file 12877_2023_3729_MOESM3_ESM.pdf]

Additional file 3. Distribution of hospitalization costs and their changing trends during 6 years

year	Medicine costs (%)	Material costs (%)	Nursing costs (%)	Examination costs (%)	General medical services costs (%)	General treatment handling costs (%)	Surgical costs (%)	Blood costs (%)	Other costs (%)
2017	41.93	11.01	3.03	20.15	5.1	7.32	3.85	1.56	6.05
2018	39.41	7.96	3.32	21.4	5.23	7.81	4	1.7	9.17
2019	35.26	10.13	3.29	23.99	4.74	7.87	4.62	1.59	8.51
2020	32.35	13.62	3.47	23.56	4.77	7.37	5.1	1.94	7.82
2021	29.79	16.55	3.05	24.57	4.16	6.72	5.06	1.84	8.26
2022	26.34	18.87	4.98	24.12	3.65	9.16	4.74	1.07	7.07
Z	-2.7649	2.4348	0.5912	0.8221	-0.5946	0.2433	0.4646	-0.1592	0.0748
P	0.0057	0.0149	0.5544	0.411	0.5521	0.8078	0.6422	0.8735	0.9404

Note: Data for 2022 only includes data from January to March.
